# Characterization of AI-2/LuxS quorum sensing system in biofilm formation, pathogenesis of *Streptococcus equi subsp. zooepidemicus*

**DOI:** 10.3389/fcimb.2024.1339131

**Published:** 2024-02-06

**Authors:** Honglin Xie, Riteng Zhang, Ruhai Guo, Yining Zhang, Jingya Zhang, Hui Li, Qiang Fu, Xinglong Wang

**Affiliations:** ^1^ College of Veterinary Medicine, Northwest A&F University, Yangling, Shaanxi, China; ^2^ School of Life Science and Engineering, Foshan University, Foshan, China

**Keywords:** *Streptococcus equi subsp. zooepidemicus*, LuxS, biofilm, capsular polysaccharide, virulence, infection

## Abstract

*Streptococcus equi subsp. zooepidemicus* (SEZ) is an opportunistic pathogen of both humans and animals. Quorum sensing (QS) plays an important role in the regulation of bacterial group behaviors. The aim of this study was to characterize the LuxS in SEZ and evaluate its impact on biofilm formation, pathogenesis and gene expression. The wild-type SEZ and its LuxS mutant (Δ*luxS*) were examined for growth, biofilm formation, virulence factors, and transcriptomic profiles. Our results showed that LuxS deficiency did not affect SEZ hemolytic activity, adhesion or capsule production. For biofilm assay demonstrated that mutation in the *luxS* gene significantly enhances biofilm formation, produced a denser biofilm and attached to a glass surface. RAW264.7 cell infection indicated that Δ*luxS* promoted macrophage apoptosis and pro-inflammatory responses. In mice infection, there was no significant difference in mortality between SEZ and Δ*luxS*. However, the bacterial load in the spleen of mice infected with Δ*luxS* was significantly higher than in those infected with SEZ. And the pathological analysis further indicated that spleen damage was more severe in the Δ*luxS* group. Moreover, transcriptomics analysis revealed significant alterations in carbon metabolism, RNA binding and stress response genes in Δ*luxS*. In summary, this study provides the first evidence of AI-2/LuxS QS system in SEZ and reveals its regulatory effects on biofilm formation, pathogenicity and gene expression.

## Introduction

*Streptococcus equi subspecies zooepidemicus* (SEZ) is a Gram positive, β-hemolytic, opportunistic pathogen that commonly resides in the upper respiratory and reproductive tracts of horses (Timoney, 2004). SEZ can also infect a broad range of mammalian hosts including pigs, ruminants, cats and dogs, causing severe disease characterized by pneumonia, septicemia and meningitis (Pelkonen et al., 2013; Jie et al., 2017; Chen et al., 2020)Virulent strains of SEZ have a history of causing severe infections in pigs, including a major outbreak in China in the 1970s that resulted in over 300,000 fatalities (Wei et al., 2009). More recently, SEZ infections with high mortality have emerged in North American swine, highlighting the threat of this bacterium (Chen et al., 2020; Song et al., 2022). Additionally, SEZ also poses zoonotic risks, causing potentially fatal diseases in humans, such as sepsis, meningitis, endocarditis, and septic arthritis (Kerdsin et al., 2022).

Quorum sensing (QS) is a common phenomenon in bacteria that allows them to communicate (Xavier and Bassler, 2003). It involves bacteria producing and detecting chemical signals in order to trigger specific responses across the bacterial population (Schauder et al., 2001). QS regulates genes involved in group behaviors that require coordination among bacteria to work effectively (Abisado et al., 2018; Wang et al., 2022). These coordinated behaviors include bioluminescence, sporulation, antibiotic production, biofilm formation, and secretion of virulence factors (Whiteley et al., 2017; Abisado et al., 2018). QS relies on bacteria secreting pheromones that accumulate in the surrounding environment. When pheromones reach a high enough concentration, they are detected by receptor proteins on the surface of bacteria. These receptor proteins activate signaling pathways inside the cell through a series of enzymatic reactions, leading to phosphorylation and activation of regulatory proteins (Jimenez and Federle, 2014). This changes the expression of specific genes, allowing bacteria to mount unified, population-level responses. By using QS, bacteria can act in a coordinated manner to perform tasks that require group effort. The QS systems identified in Streptococci can be classified into four major types: the regulator gene of glucosyltransferase (Rgg) systems, Sil systems, lantibiotic systems, and AI-2/LuxS systems (Chaussee et al., 2004; Jimenez and Federle, 2014). The Rgg family represents a group of conserved transcription factors that can sense small peptide signals and accordingly modulate downstream gene expression. In SEZ, Rgg systems have been characterized to control biofilm formation and capsular polysaccharide production (Xie et al., 2019).

The autoinducer-2 (AI-2) molecule, first found to regulate bioluminescence in *Vibrio harveyi*, is widely synthesized by the LuxS enzyme in many Gram-negative and Gram-positive bacteria, thus being proposed as a universal signal that enables interspecies cell-cell communication (Miller and Bassler, 2001). With the increasing emergence of antibiotic resistance amongst infectious bacteria, there is a dire need for alternative therapeutic strategies to control pathogens. Many studies have emphasized the functional characteristics of LuxS quorum sensing and its potential significance as a therapeutic target (Defoirdt, 2018). Currently, there are no reports on AI-2/LuxS quorum sensing in SEZ. Therefore, this study aims to clarify the role of the quorum sensing system-related gene *luxS* in SEZ by comparing and assessing its characteristics in various aspects between the wild-type SEZ strain and the LuxS deletion mutant strain.

## Results

### Characterization of the Δ*luxS* mutant strain

**Figure 1:**
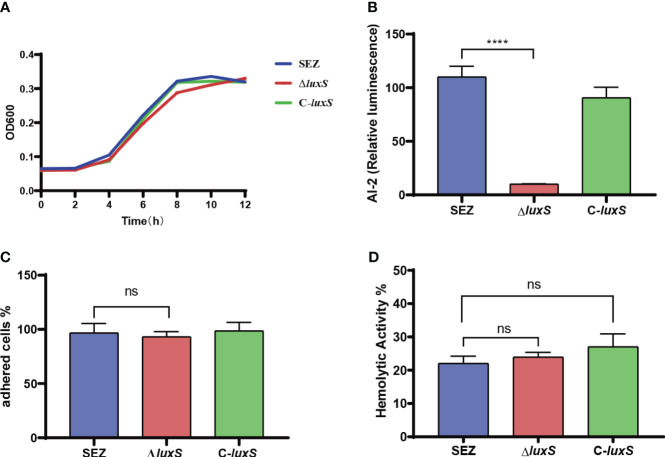
Characterization of the Δ*luxS* mutant strain. **(A)** Growth curves of the WT (wild type), Δ*luxS* (LuxS mutant), and C-*luxS* (complemented LuxS) strains in THB medium, monitored by measuring OD600. **(B)** AI-2 activity, presented as relative luminescence compared to the negative control. **(C)** Adherence of SEZ strains to PK-15 cells, assessed at 2 h post-inoculation. **(D)** Hemolytic activities of the SEZ strains, quantified using OD540 measurements in 96-well plates. Data represent mean values from three independent experiments, ns as not significant, with statistical significance denoted as ****p<0.0001.

We successfully constructed a mutation strain Δ*luxS*, and complemented strain C-*luxS*. We found that the absence of LuxS does not affect the growth of SEZ under 37°C conditions (**Figure 1A**). Then, we used the *Vibrio harveyi* BB170 as a reporter strain that responds to AI-2 by inducing luminescence (**Figure 1B**). The Δ*luxS* strain exhibited the lowest luminescence at 2 hours, indicating the functionality of the AI-2/LuxS quorum sensing system in SEZ. We compared the ability of SEZ, Δ*luxS*, and the complemented strain C*-luxS* to adhere to PK-15 cells. These results show no significant differences in adhesion among the three groups (**Figure 1C**). Furthermore, hemolysis assays revealed no differences among the three groups (**Figure 1D**).

### LuxS does not affect capsule polysaccharide production

**Figure 2:**
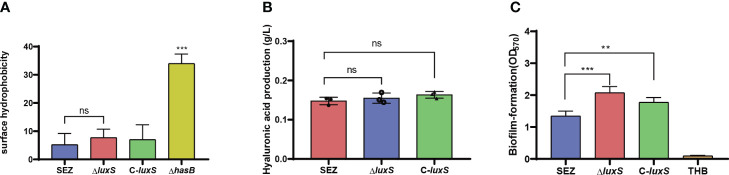
**(A)** Evaluation of surface hydrophobicity of SEZ, Δ*luxS*, C-*luxS* and capsular-deficient (Δ*hasB*) strains. **(B)** Measurement of HA production in SEZ, Δ*luxS*, and C-*luxS* strains at 10 (h) **(C)** Biofilm formation crystal violet assay comparing the WT-type SEZ, the Δ*luxS* strain and C-*luxS* cultured for 24 (h) The presented data are mean values derived from three independent experiments, ns as not significant, with statistical significance indicated as **p < 0.01,***p <0.001.

We investigated whether LuxS has an impact on hyaluronic acid (HA) production. We first assessed hydrophobicity in SEZ strains since HA is highly hydrophilic. The results indicated that WT SEZ, Δ*luxS*, and C-*luxS* strains showed no difference in hydrophobicity and the three strains exhibited very low hydrophobicity compared to the SEZ capsule-deficient strain Δ*hasB* (**Figure 2A**). Lastly, the HA production in the three groups of strains at 10 hours showed no significant differences (**Figure 2B**).

### The Δ*luxS* strain enhances biofilm formation

To investigate whether LuxS influences biofilm formation in SEZ, we initially conducted crystal violet staining using microtiter plates (**Figure 2C**). The Δ*luxS* mutant of SEZ exhibited a significantly increased biomass compared to wild-type strain. In the case of the complemented C-*luxS* strain, the biofilm content showed a reduction but still exhibited a significant difference compared to SEZ.

**Figure 3:**
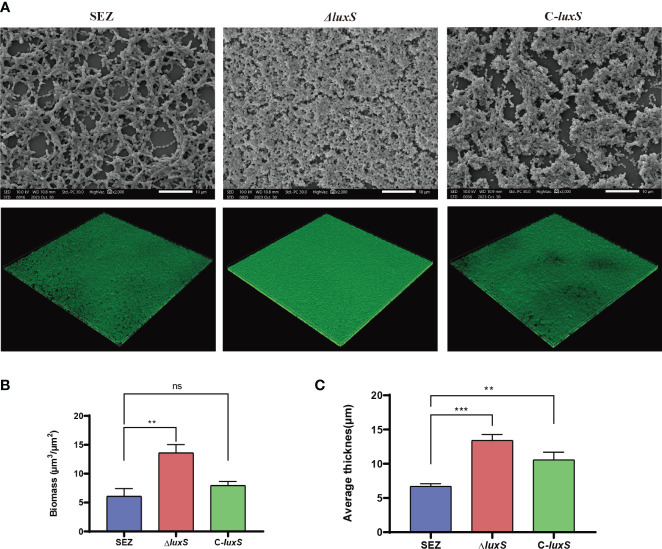
Scanning electron microscopy (SEM) and confocal laser scanning microscopy (CLSM) images of SEZ strains biofilm-formation after 48 h of incubation on glass coverslips. **(A)** SEM and CSLM image. **(B, C)** CSLM z-stack of biofilm thickness and biomass were calculated using COMSTAT 2(Vorregaard, 2008). The numbers we show are averages from three separate experiments, with **p < 0.01, or ***p < 0.001 showing where differences are significant, ns as not significant.

We further visualized biofilm formation through SEM and CLSM (**Figure 3A**). The SEM results allowed observation of the biofilm adhered to the glass surface, revealing that the biofilm formed by the Δ*luxS* mutant was comparatively denser than SEZ, while SEZ exhibited a more loosely formed biofilm.

Then, we visualized SEZ, Δ*luxS*, and C-*luxS* biofilms using CLSM, converting them into z-stacks for analysis. The biofilms were characterized for structural features, thickness, and bio-volume (**Figure 3B, C**). The Δ*luxS* strain developed a thicker mature biofilm. The application of Comstat2 for biomass and average thickness determination revealed that Δ*luxS* was significantly higher than the wild-type SEZ strain. Collectively, these results confirm that the LuxS regulates biofilm formation of SEZ.

### The Δ*luxS* strain enhances apoptosis and pro-inflammatory responses in SEZ-infected RAW264.7 cells

**Figure 4:**
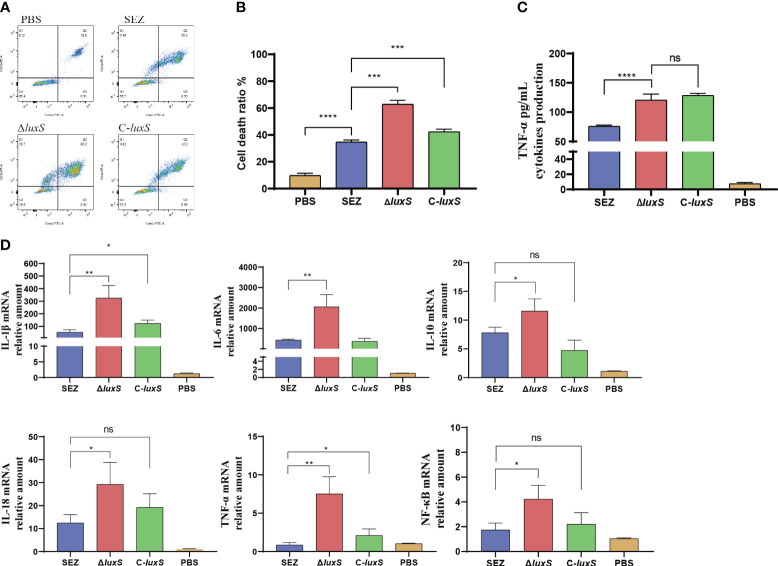
The deletion of *luxS* increases cell death and inflammatory responses in SEZ-Infected Macrophages. WT-type SEZ, Δ*luxS*, and C-*luxS* were cocultured with RAW264.7 macrophages for 12 h with MOI 1. **(A, B)** The apoptosis level of RAW264.7 macrophages was analyzed by flow cytometry. **(C)** TNF-α secretion in cell supernatant. **(D)** The mRNA expression levels of IL-1β, IL-6, IL-10, IL-18, TNF-α and transcription factor NF-κB. The data shown are average values from three independent experiments, with *p < 0.05, **p < 0.01, ***p < 0.001 or ****p < 0.0001 showing where differences are significant, ns as not significant.

Previous research has indicated that the quorum sensing system plays a significant role in immune evasion (Cao et al., 2020). To further investigate this, we conducted infection experiments using mouse macrophage RAW264.7 cells. Flow cytometry analysis revealed a significant increase in apoptosis levels in the Δ*luxS* mutant strain compared to the SEZ-infected cells (**Figures 4A, B**). The Δ*luxS* induce higher TNF-α secretion compared to SEZ, however the complemented C-*luxS* strain shows no difference compared to the Δ*luxS* (**Figure 4B**). RT-PCR analysis of pro-inflammatory cytokines, including IL-1β, IL-6, IL-10, IL-18, TNF-α, and the transcription factor NF-kB, demonstrated higher transcription levels in Δ*luxS* (**Figure 4C, D**).

### LuxS does not affect mortality in mice but alters pathologies in the spleen

**Figure 5:**
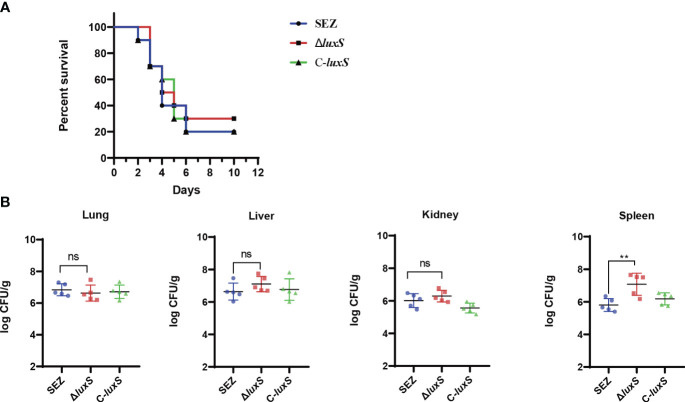
Survival rate and bacterial burden in mice infected WT SEZ, Δ*luxS*, C-*luxS* strains of 1×10^6^ CFU. **(A)** The survival rate of mice, monitored for death 12d post-infection. n=10 **(B)** Bacterial burden in lungs, livers, kidneys and spleens of the infected mice (n=5 per group). 24 h post-infection. The bacterial loads were expressed as the number of CFU/g of tissue. (*p < 0.05; ns, not significant).

**Figure 6:**
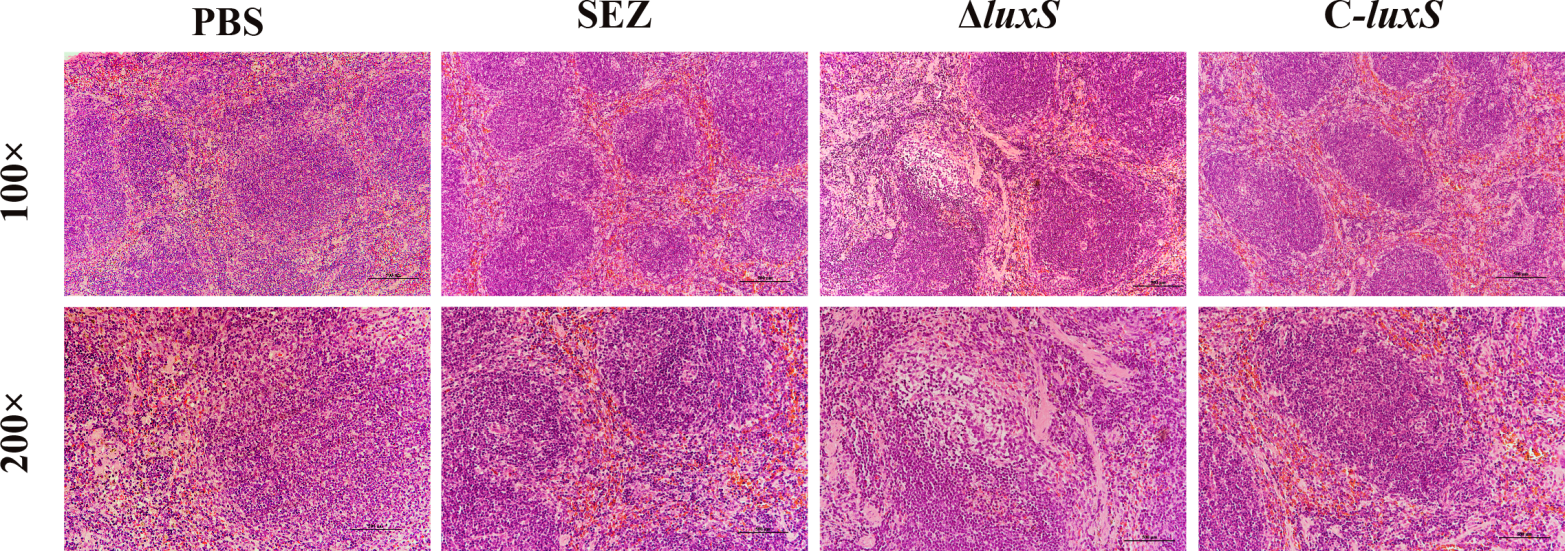
Histopathological changes observed in the spleens of mice (n=3 per group) Infected with SEZ, Δ*luxS*, and C-*luxS* strains, as revealed by H&E staining.

To investigate whether the LuxS affects the virulence of SEZ, we infected three groups of mice (n=10 for each group) via the intraperitoneal route. The final mortality of mice infected with SEZ and C-*luxS* was 80%, while Δ*luxS* group was 70% (**Figure 5A**). Then, we evaluated the bacterial burden in different organs at 24 hours post-infection. Surprisingly, the Δ*luxS* strain exhibited higher bacterial loads in the spleen compared to the SEZ wild type. There were no significant differences in bacterial loads between the Δ*luxS* strain and the SEZ wild type in the lungs, livers and kidneys (**Figure 5B**). The histopathological with H&E showed notable atrophy in the splenic nodules of the Δ*luxS* and C-*luxS* groups (**Figure 6**). The differentiation between white and red pulp was not distinct, particularly in the Δ*luxS* group, which showed more severe effects. In addition, the SEZ, Δ*luxS*, and C-*luxS* groups exhibited various levels of lymphocyte deformation and necrosis. Around these necrotic areas and within the red pulp, there was a significant infiltration of inflammatory cells, mainly neutrophils, with the Δ*luxS* group experiencing the most severe infiltration. The data suggest that while LuxS might not significantly influence the mortality rates in mice, the tissue-specific phenomena observed highlight a complex and nuanced role for LuxS in the pathogenesis and host-pathogen of SEZ.

### Transcriptomic profile of the Δ*luxS*

**Figure 7:**
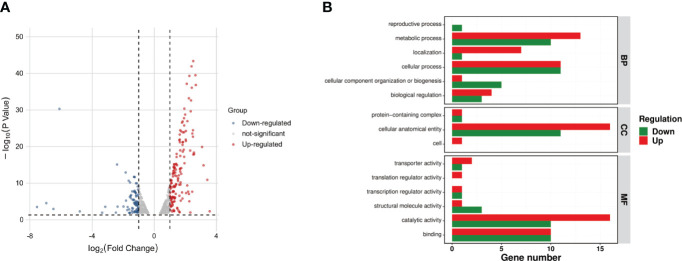
Comparative transcriptomic analysis between the SEZ wild-type and the LuxS mutant strain. **(A)**Volcano plot of differentially expressed genes between Δ*luxS* and WT SEZ groups. Red dots represent up-regulated genes; blue dots represent down-regulated genes; and gray dots represent non-differentially expressed genes. Among these, 158 genes were upregulated, and 84 genes were downregulated (FDR < 0.05); **(B)** Gene Ontology (GO) annotation analysis of differentially expressed genes. With up-regulated genes represented in red and down-regulated genes in green.

A comparative transcriptomic analysis was conducted between the SEZ wild type and the Δ*luxS* mutant strain, providing a comprehensive insight into the role of the *luxS* gene in SEZ. Among a total of 242 genes displaying differential expression in the Δ*luxS* strain compared to SEZ, 158 genes were upregulated, while 84 genes were downregulated, with an adjusted p-value (false-discovery rate [FDR]) of ≤0.05 (**Figure 7A**, **Supplementary Table 2**).

Gene ontology (GO) annotation analysis (Ashburner et al., 2000) revealed a downregulation of many genes associated with metabolic and cellular processes, while most of the downregulated genes were linked to biosynthesis, cellular localization, and structural functions in the Δ*luxS* mutant strain when compared to the SEZ (**Figure 7B**).

**Figure 8:**
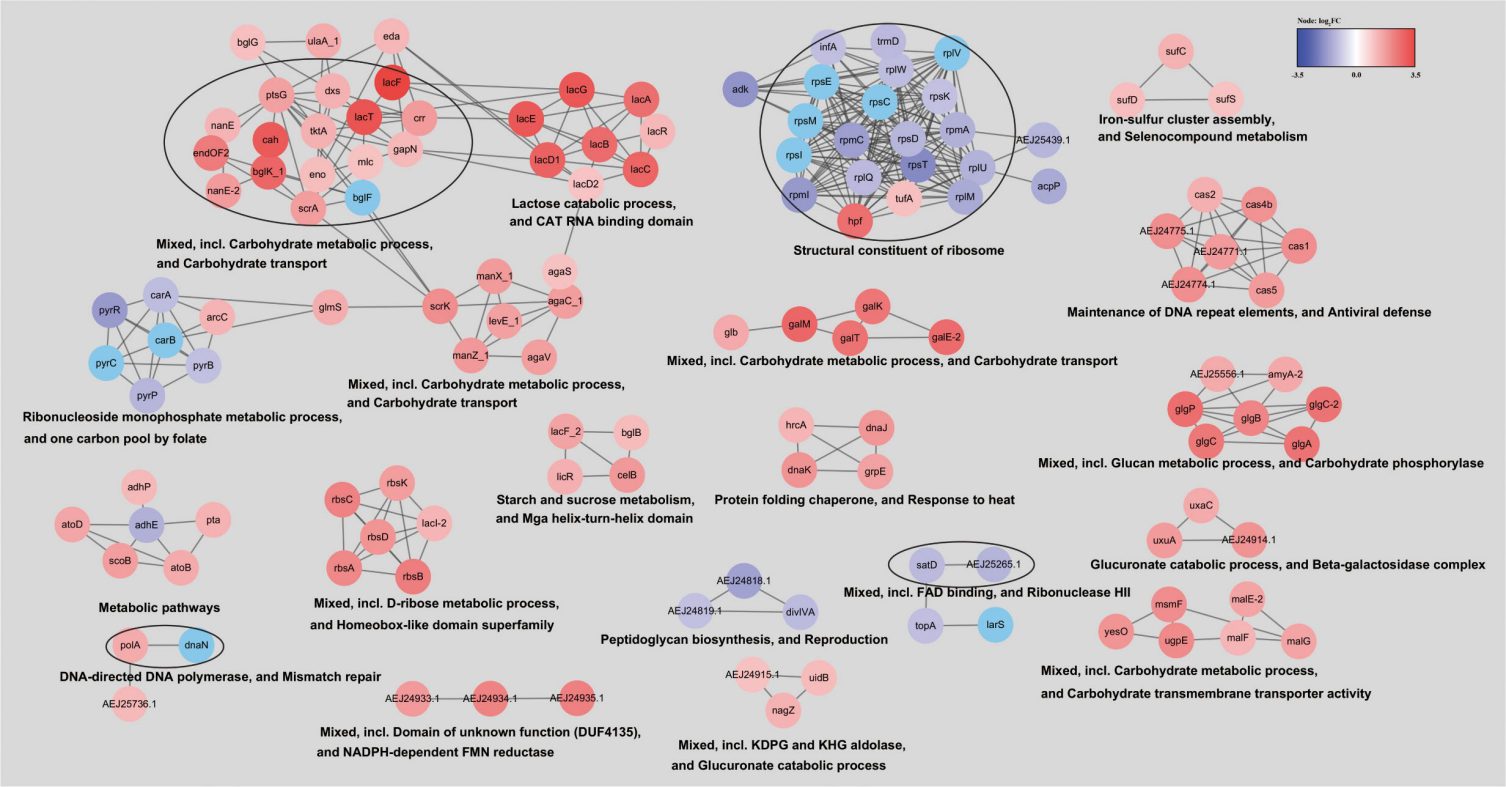
Network of differentially expressed genes in the Δ*luxS* mutant strain. To construct the network of differentially expressed genes, they were searched in a string database for SEZ ATCC 35246 organism with a 0.7 confidence (score) cutoff and a maximum of 10 additional interactors. Clustered using the MCL Cluster algorithm implemented in the Cytoscape plugin clusterMaker (Morris et al., 2011) to determine clusters and functional interactions. For each cluster, the functional enrichment was used to obtain enriched terms (available from the stringApp).

To provide a clearer understanding the differentially expressed genes in the Δ*luxS* mutant, we constructed a network (**Figure 8**). Cluster analysis showed that the majority of genes formed clusters related to carbohydrate metabolism, transport, iron-sulfur cluster and selenocompound metabolism, as well as the maintenance of DNA repeat elements and antiviral defense, all of which were upregulated. Downregulated genes were clustered into modules associated with ribonucleotide metabolism, peptidoglycan biosynthesis, DNA replication, and repair.

## Discussion

As a significant pathogen, SEZ has the potential to infect various mammals, including humans. Outbreaks in numerous animal species have been documented, and sporadic infections in humans have also been reported (Kuusi et al., 2006; Hau et al., 2022; Bosica et al., 2023; Chen et al., 2023). The exploration of immune proteins and infection mechanisms has long been a focal point of SEZ research, aiming to discover novel therapeutic approaches (Wei et al., 2013; Xie et al., 2018; Li et al., 2022; Xu et al., 2022). Many studies have shown that QS systems are responsible for group behavior, cell luminescence, antibiotic resistance, plasmid transfer, virulence factor gene expression, and biofilm formation (Waters and Bassler, 2005). Quorum sensing systems represent one of the critical targets for potential bacterial infection therapies (Bhardwaj et al., 2013; Defoirdt, 2018).

The LuxS quorum sensing system is one of the most extensively studied QS systems to date. The presence of this conserved quorum-sensing system remained unknown in SEZ. In this study, we have successfully confirmed the existence of an AI-2/LuxS quorum-sensing system in SEZ, which is capable of inducing luminescence in *Vibrio harveyi* BB170 as a reporter strain. The absence of LuxS in SEZ did not result in alterations in cell adhesion or hemolytic activity. This is primarily attributed to the fact that transcription of the hemolytic gene and the phenotypes of cell capsule remained unaltered when compared to the wild-type SEZ.

The primary component of capsular polysaccharides (CPS) in SEZ is hyaluronic acid (HA), which is produced through the hyaluronic acid synthesis (has) operon containing five genes: *hasA*, *hasB*, *hasC*, *glmU*, and *pgi* (Blank et al., 2008; Xu et al., 2016). Encapsulation by HA is recognized as a virulence factor for *Streptococcus pyogenes*, as HA can aid in protecting cells from phagocytosis and mediate the adherence of cells to host tissues(Flores et al., 2012). In SEZ, CPS is also an important virulence factor, influencing cell adhesion and phagocytosis. Our data indicate that the LuxS mutant did not alter the production of HA in SEZ.

Pathogenic bacteria exhibit an ability to form biofilms, which provide advantages for their survival against diverse stresses as well as their pathogenicity in host environments (Solano et al., 2014). The general process of biofilm formation begins with planktonic bacteria initially attaching to a surface. The bacteria then produce exopolysaccharides (EPS) that wrap around the cells, forming microcolonies. These eventually aggregate into a mature biofilm (Stoodley et al., 2002). Finally, the biofilm is dispersed to release planktonic bacteria (Kim et al., 2013). Biofilm formation is considered a virulence factor for many pathogenic bacteria. In *Streptococcus suis*, biofilms play a key role in causing persistent infections (Yi et al., 2016). In the present study, we conducted biofilm assays using crystal violet staining, SEM, and CLSM in a THB medium supplemented with fibrinogen. The Δ*luxS* mutant strain exhibited a thicker and more tightly formed biofilm, while the C-*luxS* strain, although showing a reduction, still displayed a discernible difference compared to the wild-type SEZ strain. In summary, these results collectively indicate that the absence of LuxS enhances the ability to form biofilms.

Bacteria employ a variety of strategies to evade host recognition and overcome the many challenges presented by the immune system, and quorum sensing systems are one such survival mechanism. For instance, the activation of the Rgg2/Rgg3 quorum sensing system in *Streptococcus pyogenes* can suppress NF-κB activity and reduce pro-inflammatory cytokine production in macrophages (Rahbari et al., 2021). Another study suggests that LuxS plays a role in virulence-associated traits and the immunological evasion of *Streptococcus agalactiae* (Cao et al., 2020). Our data indicates that Δ*luxS* mutation enhances some pro-inflammatory responses and promotes apoptosis in infected macrophages. Elevated pro-inflammatory cytokine expression is generally favorable for the host in clearing bacterial infections. Thought, an excessive inflammatory response can also lead to tissue damage. These results suggest that LuxS has an impact on host innate immune responses, needed further investigation into its specific mechanisms. The *luxS* gene has been previously recognized as a regulator of virulence, promoting it in pathogens like *Streptococcus suis* (Cao et al., 2011), *Haemophilus parasuis* (Zhang et al., 2019), *Edwardsiella piscicida* (Sun et al., 2020), and others (Yadav et al., 2018). However, its absence in *Haemophilus influenzae* exacerbates inflammatory responses and bacterial proliferation (Daines et al., 2005). In the case of *Staphylococcus epidermidis*, LuxS deletion paradoxically increases virulence in a rat model (Xu et al., 2006). Furthermore, in certain bacterial species such as *Porphyromonas gingivalis*, *Bacillus anthracis* and *Shigella flexneri*, LuxS does not appear to be essential for virulence (Day and Maurelli, 2001; Burgess et al., 2002; Bozue et al., 2012). Our mice experiments reveal the Δ*luxS* mutant did not significantly affect overall mortality in the mice, suggesting that it may not be an essential factor for the pathogenicity of SEZ in terms of survival outcomes. Instead, Δ*luxS* mutant strain had a notable influence on spleen bacterial burden. This organ-specific effect could be attributed to excessive tissue inflammation. To confirm this, we conducted further infections in mice and analyzed the pathological changes in their spleens, Δ*luxS* mutant showing the most significant pathologic changes. Incorporating the findings from our study, this further underscores the complex role of the LuxS quorum sensing system in modulating host-pathogen interactions.

Comparative transcriptome analysis between SEZ and the Δ*luxS* mutant underscores the broad impact of the LuxS on gene expression, affecting carbon source utilization, regulation, and metabolism in SEZ. Notably, in the Δ*luxS* strain, the LuxS transcript level was undetectable, confirming the success of the Δ*luxS* mutation. LuxS is not only responsible for AI-2 signaling molecule production but also plays a crucial role in central bacterial metabolism, including its involvement in the activated methyl cycle, as supported by our transcriptome results (Ramić et al., 2023).

Of particular note, we observed a significant up-regulation of the CRISPR-Cas (type I-C) system in the Δ*luxS* mutant (**Supplementary Figure 1**). It has been previously reported that CRISPR-Cas systems in SEZ likely play a crucial role in maintaining the stability of the bacterial genome (Waller and Robinson, 2013). Additionally, these CRISPR-Cas systems have been characterized in certain pathogenic bacteria, where they act as significant virulence factors, promoting biofilm formation and enhancing virulence (Nie et al., 2022). Cui et al. (2020) reported that CRISPR-cas3 of *Salmonella* plays a role in the regulation of QS genes, which are essential for the biofilm formation and invasion of bacteria. However, their specific effects on this bacterium have yet to be investigated and require further research. The transcript levels of *grpE*, *hrcA*, *dnaK*, and *dnaJ* were significantly up-regulated in the Δ*luxS* mutant, and these proteins are crucially involved in protein folding and the heat stress response in bacteria. They collectively play a role in regulating protein folding and cellular stress responses within bacterial cells. We conducted RT-PCR to assess the expression of these genes and evaluated their response to heat stress (**Supplementary Figure 2**). Result showed the Δ*luxS* mutant exhibited a notable resistance to heat shock (**Supplementary Figure 3**). Yi et al. (2014) employed a two-dimensional gel electrophoresis (2-DE) approach to characterize differentially expressed proteins in SEZ biofilms in comparison to their planktonic counterparts. This analysis identified 24 protein spots with varying intensities, including the upregulation of Elongation factor-Tu (EF-Tu), NADP-dependent glyceraldehyde-3-phosphate dehydrogenase (NADP-GAPDH) and glutamine–fructose-6-phosphate transaminase (*glmS*), which were also found in the Δ*luxS* mutant. It appears that we have uncovered some clues to explain the variations in biofilm formation and virulence in the Δ*luxS* mutant strain through the transcriptome data.

In summary, our study confirms the functionality of AI-2/LuxS quorum sensing in SEZ. LuxS does not impact capsule production, adhesion, or hemolysis. LuxS significantly enhances biofilm formation. While the loss of LuxS does not impact the lethality in mice, it modifies the bacterial load and the pathology in the spleen of infected animals, differences observed between SEZ and the Δ*luxS* mutant in inducing some pro-inflammatory factors and apoptosis in macrophages still reveal LuxS affects host defenses and modulates host immunity. Combined with transcriptome results, these findings provide a relatively comprehensive characterization of the role of LuxS in SEZ to date.

## Materials and methods

### Bacterial strains, plasmids, culture conditions

The bacterial strains and plasmids used in this study are detailed in (**Supplementary Table 1**). The SEZ ATCC35246 bacterial strain, originally isolated from an infected pig in Sichuan (Ma et al., 2011), was preserved in our laboratory for research purposes. SEZ strain were grown in Todd–Hewitt broth (THB) at 37°C. *Vibrio harveyi* strains BB170 were used as biosensors of the AI-2 signal and as the positive control for AI-2 bioassay, respectively. The *V. harveyi* strains were cultured in autoinducer bioassay medium (AB) as described (Bassler et al., 1993). The RAW264.7 macrophages and PK-15 cells were also maintained in our laboratory. These cell lines were cultured in DMEM (Gibco, USA), supplemented with 10% fetal bovine serum (FBS), and maintained in a humidified 5% CO_2_ incubator at 37°C.

### Construction of the LuxS mutant strain and complemented strain

The deletion of the *luxS* gene in SEZ was conducted following a previously established protocol (Takamatsu et al., 2001; Wei et al., 2012b). In sum, we initiated the process by using PCR amplification to generate upstream and downstream fragments of the *luxS* gene with specific primers (**Supplementary Table 1**). Then, these PCR products were ligated into the temperature-sensitive suicide vector, pSET-4s, using appropriate restriction enzymes. The resulting plasmid, denoted as pset4s-*luxS*, was introduced into SEZ through electroporation.

Bacterial cultures were under 28°C in the presence of 100 μg/mL spectinomycin (Spc). When the cultures reached the mid-logarithmic growth phase, bacteria were sub-cultured in THB supplemented with 100μg/mL Spc and further incubated at 28°C until they reached the early logarithmic phase. The culture was then shifted to 37°C for a duration of 4 hours. Subsequently, cells were plated on TSA plates and kept at 28°C. Colonies that did not exhibit resistance to Spc at 37°C were selected, indicating the loss of spectinomycin resistance conferred by the plasmid. Confirmation of the double-crossover homologous recombination mutant, designated as Δ*luxS*, was carried out using PCR analysis with primers luxSL-F and luxSR-R.

As for the complemented strain C-*luxS*, we amplified fragments that included the promoter region and the *luxS* gene, carried out the necessary digestion, and incorporated them into the pSET2 vector. This resulting construct was then introduced into the Δ*luxS* strain through electroporation to achieve complementation.

To assess the growth characteristics of SEZ, the Δ*luxS* mutant, and C-*luxS*, a single colony from each strain was cultured overnight at 37°C and adjusted to an OD600 of 0.3 using fresh THB medium. Subsequently, the cells were inoculated into fresh THB medium at a 1:1000 ratio and incubated at 37°C with shaking at 180 rpm for 24 hours. Cell densities were measured every 2 hours at OD_600_.

### AI-2 bioassay

For AI-2 detection, established protocols were followed (Buck et al., 2009). The SEZ strain, Δ*luxS*, and the complemented C-*luxS* were cultured in THB medium until they reached an optical density of 0.3 at 600 nm. To obtain cell-free culture fluid (CF), the supernatant was filtered through a 0.22-μm-pore-size filter (Millipore, Bedford, MA). The reporter strain *V. harveyi* BB170 was diluted at a ratio of 1:5,000 in autoinducer bioassay (AB) medium, and AB medium was added to the diluted BB170 culture at a 1:10 ratio (vol/vol). This mixture was then incubated at 28°C. Aliquots of 100μL were dispensed into white, flat-bottomed, 96-well plates for AI-2 activity detection. CFs from BB170 and AB medium served as positive and negative controls, respectively. Luminescence measurements were conducted using a microplate reader.

### Cell adhesion and hemolysis assays

To investigate the impact of LuxS on SEZ adhesion to cells, we conducted the following procedure (Tang et al., 2017). Cultured bacterial strains (SEZ, Δ*luxS*, C-*luxS*) were harvested, washed with sterile PBS, and then added to PK15 cells cultured in a 12-well dish at a multiplicity of infection (MOI) of 10. The cell-bacteria mixtures were co-incubated for 2 hours. Following incubation, non-adherent bacteria were removed by rinsing the cells with PBS. Subsequently, the cells were resuspended in sterile water, appropriately diluted, and plated onto tryptic soy agar (TSA) plates for colony counting.

To assess hemolysin activity in the Δ*luxS* mutant, we conducted a hemolytic assay following a previously described protocol (Jusuf et al., 2021). In brief, bacterial cells were washed and resuspended in PBS. Approximately 10^6^ bacterial cells in 100 μL were added to a 96-well microtiter plate. Fresh pig blood was processed to remove serum and washed with physiological saline 2-3 times, then diluted to a 5% concentration, with 100 μL added to each well. The plate was incubated for 2 hours at 37°C. After incubation, the supernatant was collected following centrifugation, and the absorbance was measured at 420 nm. A 0.1% Triton X solution was used as a positive control to calculate the percentage of bacterial hemolysis activity.

### Hydrophobic and HA production assay

To assess the hydrophobic properties of the bacteria, an equal volume of bacterial suspension was combined with xylene and mixed for 2 minutes. The mixture was then left to stand for 30 minutes, allowing the phases to separate, after which the OD_600_ of the aqueous phase was measured. The hydrophobicity percentage (H%) was calculated using the formula H% = [(H0 − H)/H0] × 100%, where H0 represents the initial absorbance before mixing with xylene, and H represents the absorbance after xylene extraction (Del Re et al., 2000). The SEZ capsule-deficient strain Δ*hasB* was used as the control group in this assay.

The cell-associated hyaluronic acid (HA) of SEZ was measured using a procedure inspired by existing methods, with some modifications (Velineni and Timoney, 2015). In brief, 10 ml of SEZ culture in THB, grown at 37°C, was centrifuged at 8000 g for 10 minutes. The resulting pellet was gently washed with PBS and then resuspended in 2 ml of distilled water. This suspension was transferred to a capped glass tube, to which 5 ml of chloroform was added. The mixture was then vigorously mixed for 1 hour. Following this, the sample was centrifuged at 8,500 g for 10 minutes, and the aqueous phase was carefully transferred to a new container. To this aqueous phase, two volumes of cetyltrimethylammonium bromide (CTAB) buffer (2.5 g L^−1^) were added, and the mixture was gently stirred. The reaction was allowed to proceed for 10 minutes at room temperature, after which the OD_600_ was measured (Xie et al., 2019). The concentration of HA in the sample was determined based on the OD600 value and a standard curve. This standard curve was established using a stock solution of HA prepared with standard HA (Sigma, Shanghai, China).

### Biofilm-formation assay

Biofilm formation was quantified by the crystal violet assay (Bonifait et al., 2008; Wang et al., 2011). Bacterial cultures were grown until they reached an optical density (OD600) of approximately 0.3. Then, 10 μL of the bacterial suspension was added to 96-well flat-bottomed plastic culture plates containing 90 μL of THB supplemented with 10 mg/mL of fibrinogen. The plates were incubated at 37°C for 24 hours, with each bacterial strain tested in triplicate. The culture medium was discarded, and the wells were gently washed three times with sterile PBS to remove loosely adherent cells. The remaining bacteria attached to the wells were fixed with methanol for 30 minutes and air-dried. A 1% crystal violet solution was added to each well, and the plates were stained for 10 minutes at room temperature. Excess crystal violet was removed and the wells were rinsed multiple times with distilled water. The biofilm-associated crystal violet was then solubilized with 100 μL of 95% (v/v) ethanol, and the optical density at 570 nm was measured using a microplate reader.

For scanning electron microscopy (SEM), SEZ was inoculated into a 24-well culture plate containing sterile glass slides and incubated at 37°C for 48h. The slides were then removed, washed three times with sterile PBS, and fixed in a solution of 5% glutaraldehyde, 4.4% formaldehyde, and 2.75% picric acid in 0.05% sodium cacodylate buffer for at least 1 h. The sample underwent sequential dehydration with 25%, 50%, 70%, 80%, and 95% ethanol. SEM was performed as previously described (Petruzzi et al., 2017), using a scanning electron microscope.

To study biofilms using confocal laser scanning microscopy (CLSM), each biofilm was stained with 0.3% SYTO-9 (obtained from Sigma-Aldrich, China) and incubated in darkness for 15 minutes for fluorescent labeling. The analysis of the biofilm images was conducted using Zeiss confocal software, enabling the acquisition of z-stack images. The thickness and biovolume of the biofilms were quantified using the COMSTAT2 software available at www.comstat.dk.

### RAW264.7 macrophages infection

In the experiment with macrophages, we used RAW264.7 cells and followed a method that was a bit different from what others have done before. We put the macrophages in six-well plates. After that, we added SEZ bacteria to 12-well plates with a MOI of 1, and co-incubate for 12 h. For the negative control, we just added 100 μL of PBS.

To evaluate apoptosis, the treated cells were collected, washed twice with ice-cold PBS, and then resuspended in 1× Annexin V binding buffer at a concentration of 1×10^6^ cells/mL. Apoptosis detection was carried out using the Annexin V-FITC Apoptosis Detection Kit (Sigma, Shanghai, China), strictly adhering to the manufacturer’s instructions. The stained cells were analyzed via flow cytometry within 1 hour of staining.

The cell supernatant was collected by centrifugation at 3,000g, and the secretion level of TNF-α was determined using an ELISA assay kit (Enzyme-linked Biotechnology, Shanghai, China).

Quantitative real-time reverse-transcription polymerase chain reaction (qRT-PCR) was used to assess the transcription levels of pro-inflammatory cytokines (IL-1β, IL-6, IL-10, IL-18, TNF-α) and NF-κB. Briefly, post-infection, the cells were washed twice with sterile PBS. Total RNA was extracted using TRIzol reagent (Sigma, Shanghai, China), followed by cDNA synthesis using the Takara reverse transcription kit. Real-time quantitative PCR was performed using SYBR Green Premix (Takara, Beijing, China) on a LightCycler 480 fast real-time cycler. Fold changes were calculated using the 2^-ΔΔCt^ formula (Livak and Methods, 2000). The data are presented as the means ± standard deviation (SD) of triplicate reactions for each gene transcript, and the primer sequences are provided in (**Supplementary Table 1**).

### Mice survival curve and tissue bacterial load

Four-to-six-week-old BALB/c mice were obtained from Beijing Vetonlitech Experimental Animal Technology Co. Bacterial suspensions were prepared by diluting the bacteria in PBS, 1 × 10^6^ CFU of bacteria were administered via intraperitoneal injection.

Tissue bacterial burden assays were conducted as previously described (Wei et al., 2012a). Briefly, mice were euthanized 24 hours after infection, and their spleens, livers, kidneys, and lungs were collected and weighed. Then, the tissues were homogenized and diluted in sterile saline (g/mL). The homogenized tissue samples were plated onto TSA plates for culturing, and the colonies were counted.

### Histological analysis

Briefly, the mice were infected with 1 × 10^6^ CFU of bacteria. At 24 h post-infection, the infected mice were euthanized, and the spleen were collected and placed into 10% neutral buffered formalin. After fixation, the tissues were dehydrated and embedded in paraffin, and 4-mm sections were taken and stained with hematoxylin and eosin (H&E) for histological evaluation.

### Transcriptomic analysis

The Δ*luxS* mutant and wild-type strains were incubated in THB on a shaker at 37°C for 10 hours. After incubation, the cells were harvested by centrifugation at 6,000 g for 10 minutes at 4°C. Total RNA samples were extracted using the TRIzol method. RNA sample quality was assessed using Thermo NanoDrop One and Agilent 4200 Tape Station. Once qualified, the ribosomal RNA (rRNA) was removed using the Epicentre Ribo-Zero rRNA Removal Kit.

Library preparation followed a standardized protocol using the NEBNext Ultra II Directional RNA Library Prep Kit for Illumina, involving mRNA fragmentation, cDNA synthesis, double-stranded cDNA generation, purification, enzymatic degradation, end repair, adapter ligation, fragment size selection, and final PCR amplification. Libraries meeting quality control criteria underwent PE150 sequencing on the Illumina platform. Raw sequencing data were processed into FASTQ files, including read sequences and corresponding quality scores. The data sets generated for this study can be found in the NCBI Sequence Read Archive (SRA) repository under BioProject accession number PRJNA1048089.

Before conducting the differential expression analysis, we conducted quality control on the sequencing reads and trimmed the adapter sequences. The sequencing reads from each library were then used for differential expression analysis with the read counts. As a reference genome sequence, we used the complete genome sequence of *Streptococcus equi subsp. zooepidemicus* (ATCC_35246) and its genome annotation from the NCBI nucleotide database (accession number: NC_017582.1).

To compare gene expression between SEZ and Δ*luxS*. Genes with absolute log2 fold change values of ≥1 and false discovery rate (FDR) P values of ≤0.05 were considered differentially expressed. For further analysis of these differentially expressed genes, we utilized the STRING Consortium 2020, which provides functional enrichment analysis of protein-protein interaction networks through the STRING mapper tool (Szklarczyk et al., 2023).

### Data analysis

The data in this study are expressed as means ± standard deviation (SD). For the statistical analysis, we used two-way ANOVA, conducted with Graph Pad Prism 9.0 (GraphPad Software Inc., USA). We marked significant differences with asterisks: * for *p* < 0.05, ** for *p* < 0.01, and *** for *p* < 0.001. Different letters represent significant differences within each strain, with *p* < 0.05 as the threshold for significance. All data are presented as means ± standard error of the mean (SEM), and the results are compiled from a minimum of three independent experiments.

## Data availability statement

The original contributions presented in the study are included in the article/**Supplementary Material**. Further inquiries can be directed to the corresponding author.

## Ethics statement

The animal study was approved by All operations in this study involving animal experiments were handled according to the Ethics Committee at Northwest A&F University (approval number DY2022009). The study was conducted in accordance with the local legislation and institutional requirements.

## Author contributions

HX: Conceptualization, Data curation, Formal analysis, Investigation, Methodology, Software, Supervision, Visualization, Writing – original draft, Writing – review & editing. RZ: Data curation, Formal analysis, Software, Visualization, Writing – review & editing. RG: Investigation, Methodology, Writing – review & editing. YZ: Conceptualization, Formal analysis, Writing – review & editing. JZ: Project administration, Writing – review & editing. HL: Formal analysis, Writing – review & editing. QF: Resources, Validation, Writing – review & editing. XW: Funding acquisition, Project administration, Resources, Validation, Writing – review & editing.
