# Supplementary material for: Characterization of AI-2/LuxS quorum sensing system in biofilm formation, pathogenesis of Streptococcus equi subsp. zooepidemicus
Source: Front Cell Infect Microbiol. 2024 Feb 6;14:1339131. doi: 10.3389/fcimb.2024.1339131 (PMC10876813; doi:10.3389/fcimb.2024.1339131)
Supplement: Supplementary file 1 [file Table_1.docx]

| **Table 2** | | |
| --- | --- | --- |
| **Bacterial strains and plasmids used in this study.** | | |
| **Plasmid or strain** | **Relevant characteristics** | **References; character** |
| **PASMIDS** |  |  |
| pSET4s | S. suis thermosensitive suicide vector; Spc^R^ | (Takamatsu et al., 2001b) |
| pSET2 | E. coli–S. suis shuttle vector; Spc^R^ | (Takamatsu et al., 2001a) |
| pMD19-T | Clone vector | Takara |
| **STRAINS** |  |  |
| SZE | S. *equi* *subsp.* *zooepidimicus* ATCC35246, wild type strain | (Ma et al., 2011) |
| Δ*luxS* | Mutation in *luxS* gene of SEZ | this study |
| C-*luxS* | Complemented strain of Δ*luxS*;Spc^R^ | this study |
| Δ*hasB* | The SEZ capsule-deficient strain | (Wei et al., 2012) |
| *V. harveyi* BB170 | AI-2 Signal fluorescence detection | (Bassler et al., 1993) |
| Spc^R^, spectinomycin resistant | | |
| **Primers used in this study.** | | |
| **Primer** | **Sequence (5′–3′)** | **character** |
| **Construction of the luxS mutant and complementation strain** | | |
| luxSL-F | TTTAGTCGACAGATTTTCATCCTTA | Restriction site：SAII |
| luxSL-R | TAGATTTTAGAAAAATGTGGATCCATT | Restriction site：BamHI |
| luxSR-F | CAATTGGATCCTCGCTTTTCTGCTAA | Restriction site： BamHI |
| luxSR-R | TGGGAATTCAGGCTATGACCCTTCA | Restriction site： EcoRI |
| C-luxS-R | GGATCCCAGACTTGAGTCTAATCGAAATAAG | Restriction site：BamHI |
| C-luxS-R | GAATTCCTATTATTATGAGGTGACATGGCGGTTA | Restriction site: EcoRI |
| **Real-time PCR in RAW264.7 cells** | | |
| IL-1β-F | GCAACTGTTCCTGAACTCAACT | NCBI Reference Sequence: NM_008361.4 |
| IL-1β-R | ATCTTTTGGGGTCCGTCAACT |  |
| IL-6-F | TAGTCCTTCCTACCCCAATTTCC | NCBI Reference Sequence: NM_031168.2 |
| IL-6-R | TTGGTCCTTAGCCACTCCTTC |  |
| TNF-α-F | CCTGTAGCCCACGTCGTAG | NCBI Reference Sequence: NM_013693.3 |
| TNF-α-R | GGGAGTAGACAAGGTACAACCC |  |
| GAPDH-F | TGGCCTTCCGTGTTCCTAC | NCBI Reference Sequence: NM_008084.3 |
| GAPDH-R | GAGTTGCTGTTGAAGTCGCA |  |
| IL-18-F | GACTCTTGCGTCAACTTCAAGG | NCBI Reference Sequence: NM_008360.2 |
| IL-18-R | CAGGCTGTCTTTTGTCAACGA |  |
| NF-κB-F | AGGCTTCTGGGCCTTATGTG | NCBI Reference Sequence: NM_008689.3 |
| NF-κB-R | TGCTTCTCTCGCCAGGAATAC |  |
| IL-10-F | GCTCTTACTGACTGGCATGAG | NCBI Reference Sequence: NM_010548.2 |
| IL-10-R | CGCAGCTCTAGGAGCATGTG |  |
| **Real-time PCR verification of differentially expressed genes** | | |
| 16SrRNA-R | ATCCGAACTGAGATTGGC | |
| 16SrRNA-F | CCCTTATGACCTGGGCTA | |
| LuxSrRNA-F | CGACTTGTTCAGCCTAAT | |
| LuxSrRNA-R | GCTTGGCTAATAAATGCTC | |
| hcrArRNA-F | TCGTGATTTGCTGGTGCT | |
| hcrArRNA-F | CTGAGGGATTTCGGTTCG | |
| grprRNA-F | GCGTGCTTTAGCTGTTGA | |
| grpErRNA-F | GCTGGTAGGGTTTGGACT | |
| dnaKrRNA-F | AGTTGTAGCGATGGGTGC | |
| dnaKrRNA-F | GTGTTGCGGTCAATAAGC | |

Bassler B L, Wright M, Showalter R E, Silverman M R (1993). Intercellular signalling in Vibrio harveyi: sequence and function of genes regulating expression of luminescence. Mol Microbiol, 9(4): 773-786

Ma Z, Geng J, Zhang H, Yu H, Yi L, Lei M, Lu C P, Fan H J, Hu S (2011). Complete genome sequence of *Streptococcus equi* *subsp.* *zooepidemicus* strain ATCC 35246. J Bacteriol, 193(19): 5583-5584

Takamatsu D, Osaki M, Sekizaki T (2001a). Construction and characterization of *Streptococcus suis*-*Escherichia coli* shuttle cloning vectors. Plasmid, 45(2): 101-113

Wei Z, Fu Q, Chen Y, Cong P, Xiao S, Mo D, He Z, Liu X. The capsule of *Streptococcus equi* ssp. *zooepidemicus* is a target for attenuation in vaccine development. Vaccine. 2012 Jun 29;30(31):4670-5.

Takamatsu D, Osaki M, Sekizaki T (2001b). Thermosensitive suicide vectors for gene replacement in *Streptococcus suis*. Plasmid, 46(2): 140-148

Wei Z, Fu Q, Chen Y, Cong P, Xiao S, Mo D, He Z, Liu X (2012). The capsule of *Streptococcus equi* ssp*. zooepidemicus* is a target for attenuation in vaccine development. Vaccine, 30(31): 4670-4675
