# Supplementary material for: Characterization of AI-2/LuxS quorum sensing system in biofilm formation, pathogenesis of Streptococcus equi subsp. zooepidemicus
Source: Front Cell Infect Microbiol. 2024 Feb 6;14:1339131. doi: 10.3389/fcimb.2024.1339131 (PMC10876813; doi:10.3389/fcimb.2024.1339131)
Supplement: Supplementary file 3 [file Image_1.pdf]

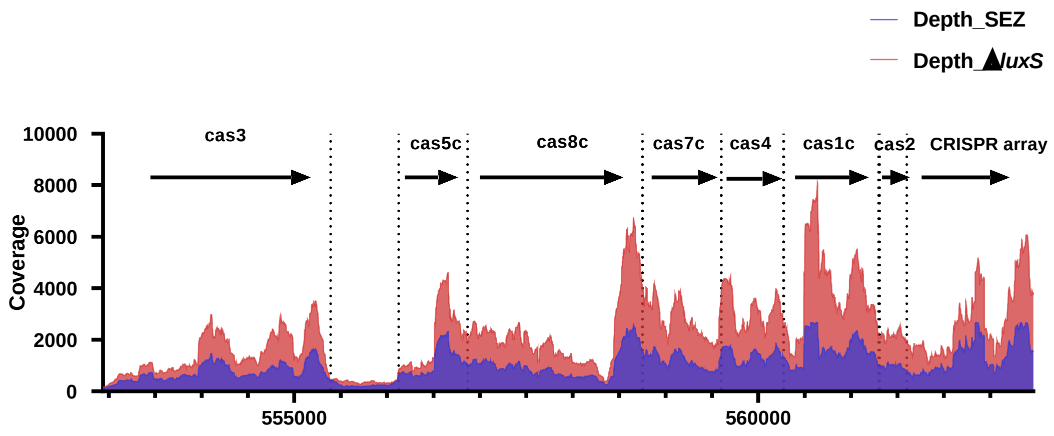

Figure S1 RNA-Seq data showing the transcriptional coverage of the type I-C CRISPR in WT SEZ and Δ*luxS* starins.
