# Supplementary material for: Characterization of AI-2/LuxS quorum sensing system in biofilm formation, pathogenesis of Streptococcus equi subsp. zooepidemicus
Source: Front Cell Infect Microbiol. 2024 Feb 6;14:1339131. doi: 10.3389/fcimb.2024.1339131 (PMC10876813; doi:10.3389/fcimb.2024.1339131)
Supplement: Supplementary file 4 [file Image_2.pdf]

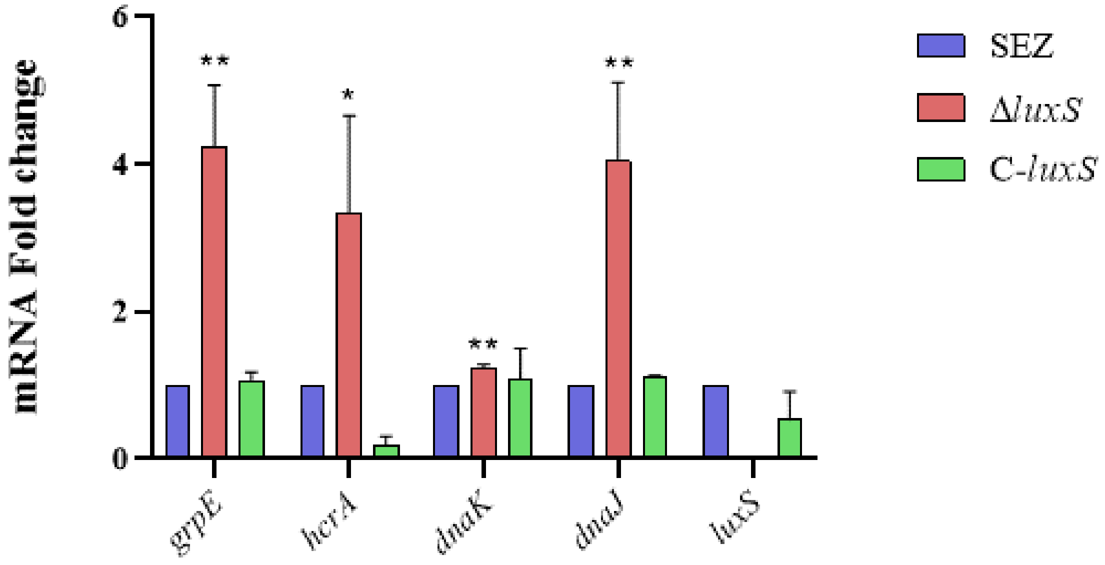

Figure S2 RT-qPCR verification. Four proteins are crucially involved in protein folding and the heat stress response and *luxS*. 16S rRNA was amplified as endogenous control and the results were analyzed using the $2^{-\Delta\Delta Ct}$ method in triplicates in three independent experiments. Data represent mean values from three independent experiments, with statistical significance denoted as *$p < 0.05$, **$p < 0.01$.
