# Supplementary material for: Characterization of AI-2/LuxS quorum sensing system in biofilm formation, pathogenesis of Streptococcus equi subsp. zooepidemicus
Source: Front Cell Infect Microbiol. 2024 Feb 6;14:1339131. doi: 10.3389/fcimb.2024.1339131 (PMC10876813; doi:10.3389/fcimb.2024.1339131)
Supplement: Supplementary file 5 [file Image_3.pdf]

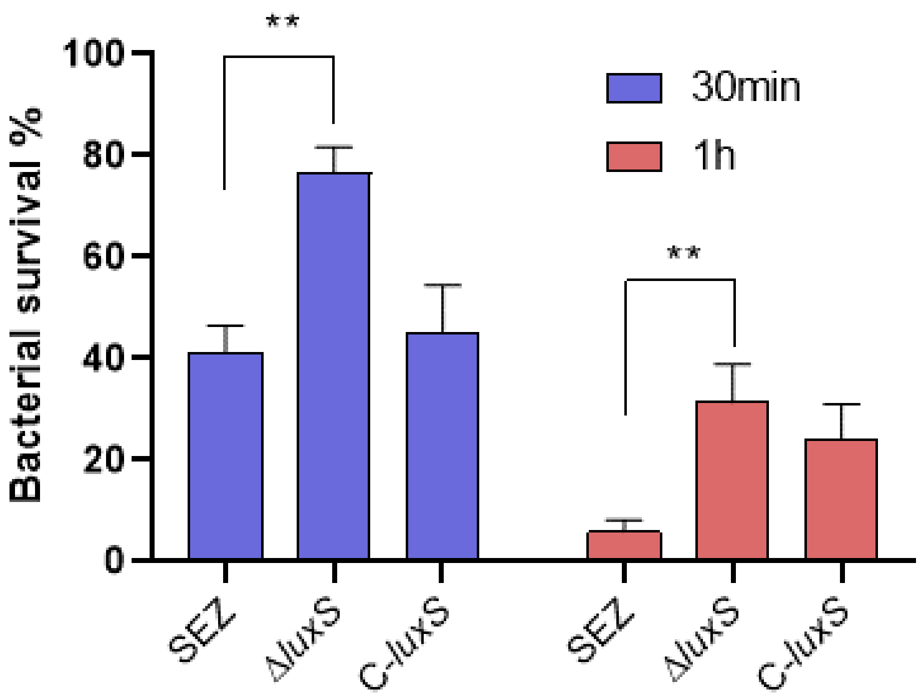

Figure S3 The survival rates of SEZ, Δ*luxS*, C-*luxS*, strains under 48 °C heat shock conditions for 30min or 1h. The method is as previously reported (Zhang et al., 2022). Data represent mean values from three independent experiments, with statistical significance denoted as $**p < 0.01$.

1. Zhang B, Jiang C, Cao H, Zeng W, Ren J, Hu Y, Li W, He Q. Transcriptome analysis of heat resistance regulated by quorum sensing system in *Glaesserella parasuis*. Front Microbiol. 2022 Aug 11;13:968460.
